# The Use of an IL-1 Receptor Antagonist Peptide to Control Inflammation in the Treatment of Corneal Limbal Epithelial Stem Cell Deficiency

**DOI:** 10.1155/2015/516318

**Published:** 2015-02-01

**Authors:** E. Fok, S. R. Sandeman, A. L. Guildford, Y. H. Martin

**Affiliations:** ^1^Biomaterials and Medical Devices Research Group, School of Pharmacy and Biomolecular Sciences, University of Brighton, Brighton BN2 4GJ, UK; ^2^Blond McIndoe Research Foundation, Queen Victoria Hospital, East Grinstead RH19 3DZ, UK; ^3^Brighton Studies in Tissue-Mimicry and Aided Regeneration, School of Pharmacy and Biomolecular Sciences, University of Brighton, Brighton BN2 4GJ, UK

## Abstract

Corneal limbal stem cell deficiency (LSCD) may be treated using *ex vivo* limbal epithelial stem cells (LESCs) derived from cadaveric donor tissue. However, continuing challenges exist around tissue availability, inflammation, and transplant rejection. Lipopolysaccharide (LPS) or recombinant human IL-1*β* stimulated primary human keratocyte and LESC models were used to investigate the anti-inflammatory properties of a short chain, IL-1 receptor antagonist peptide for use in LESC sheet growth to control inflammation. The peptide was characterized using mass spectroscopy and high performance liquid chromatography. Peptide cytotoxicity, patterns of cell cytokine expression in response to LPS or IL-1*β* stimulation, and peptide suppression of this response were investigated by MTS/LDH assays, ELISA, and q-PCR. Cell differences in LPS stimulated toll-like receptor 4 expression were investigated using immunocytochemistry. A significant reduction in rIL-1*β* stimulated inflammatory cytokine production occurred following LESC and keratocyte incubation with anti-inflammatory peptide and in LPS stimulated IL-6 and IL-8 production following keratocyte incubation with peptide (1 mg/mL) (*P* < 0.05). LESCs produced no cytokine response to LPS stimulation and showed no TLR4 expression. The peptide supported LESC growth when adhered to a silicone hydrogel contact lens indicating potential use in improved LESC grafting through suppression of inflammation.

## 1. Introduction

Approximately 4.9 million people worldwide are bilaterally blind due to corneal opacity and corneal blindness is the fifth most common cause of blindness globally [1]. The restoration of a healthy limbal epithelial stem cell fraction is vital to the treatment of corneal blindness associated with the breakdown of corneal epithelial integrity. Limbal epithelial stem cells (LESCs) are located in the basal region of the limbus, where the corneal epithelium meets the sclera, and are important in maintaining the structural integrity and transparency of the cornea [2]. LESCs are responsible for maintenance of corneal epithelial integrity through provision of a continuously renewed corneal epithelium, by producing a steady supply of daughter transient amplifying cells which differentiate into basal, wing, and squamous epithelial cells [3]. Cells move in an inwardly spiraling pattern from the basal to apical layers of the epithelium where the squamous cells at the surface of the cornea are continuously lost into the tear film. LESCs may be destroyed by injury (chemical burns, contact lens-induced keratopathy), infection or disease (aniridia, Stevens-Johnson syndrome, and ocular cicatricialpemphigoid) [4–7] resulting in a condition known as limbal epithelial stem cell deficiency (LSCD). When this occurs, conjunctival epithelial overgrowth, vascularisation, and chronic inflammation may result in scarring and the loss of corneal transparency.

Therapeutic replacement of corneal limbal epithelial stem cells is an ongoing area of investigation. In the UK, the most widely used surgical therapy to treat LSCD is the use of cadaveric-derived LESC cultures which are grown to confluency and then transplanted using an amnion bandage. The use of cadaveric tissue is thought to reduce the risk of tissue rejection since a number of human leukocyte antigen-DR (HLA-DR) expressing Langerhans cells are significantly reduced following 14 days of storage [8, 9]. However, immunosuppressant drugs are still required after treatment and the rate of LESC allograft failure after 6 months is approximately 27% and is often associated with chronic inflammation [10–12].

Current techniques to control inflammation rely on the use of anti-inflammatory drugs or amniotic membrane. Amnion is used as both a bandage and as a LESC sheet carrier membrane and is thought to possess anti-inflammatory properties. Although the exact mechanisms are unknown, amniotic membrane-derived epithelial cells have been shown to express IL-1 receptor antagonist (IL-1ra) [13] and corneal epithelial cells cultured on amniotic membrane produced reduced quantities of IL-1 [14]. IL-1 is known to instigate corneal inflammation and angiogenesis so that suppression of its activity may facilitate graft integration. The amniotic membrane is thought to provide a favourable microenvironment for LESC similar to the corneal limbus [15, 16]. However, the use of anti-inflammatory drugs and amnion is associated with significant clinical risk including interdonor variability, increased risk of infection, and corneal melting [17]. Amniotic membrane is derived from the inner placenta and, like other tissues used in transplantation, carries a risk of viral pathogen transmission [18–20]. Furthermore, the application of amniotic membrane in clinic requires the use of sutures or fibrin glue, which introduces additional risk of infection and irritation [21, 22]. Corneal calcification, linked to the use of amnion in conjunction with eye drops after surgery, can also result in corneal clouding caused by insoluble deposits and further surgery is often required. Such issues highlight the benefit of developing alternative approaches to the management of inflammation which utilises the positive aspects of amnion function without the negative side effects of amnion use. Other carrier materials, including fibrin matrices, Mebiol gel polymers, recombinant human cross-linked collagen scaffolds, collagen gel, keratin film, silk fibroin, and temperature-responsive polymers, have been investigated as amnion substitutes [23–30]. However, none of these has been widely used in the clinical setting and long-term clinical outcomes remain unknown.

Key issues therefore remain in the control of inflammation and in the optimisation of cell carrier technology for use in the transport and application of cell grafts. The aim of this study was to develop an anti-inflammatory peptide which can be combined with a carrier matrix to facilitate cell grafting and concurrently reduce inflammation. Here we report the synthesis and characterisation of a short sequence IL-1 beta antagonist peptide as an anti-inflammatory amnion biomimic. Using a cell model of corneal inflammation, we show a reduction in proinflammatory cytokines IL-6 and IL-8 with the peptide when cells are stimulated with LPS or IL-1*β*. Use of a short chain rather than full length peptide improves the potential for conformational binding of active peptide to a carrier surface. We further show that the peptide can be conjugated to a commercially available silicon hydrogel contact lens, while maintaining corneal limbal stem cell growth.

## 2. Materials and Methods

All reagents were purchased from Sigma-Aldrich, UK, unless stated otherwise.

### 2.1. Synthesis of the IL-1 Receptor Antagonist Peptide and Isolation and Growth of Primary Corneal Epithelial Cells and Keratocyte Cell Strains for Cell Assays

The study utilised an IL-1 receptor antagonist (ra) peptide first identified by Yanofsky et al. [31] and Vigers et al. [32] with NCBI reference AF10847 and primary sequence of COOH–LPLAYPQWYYANSEEWTFPTE–NH_2_. Peptide chain assembly was carried out using solid phase peptide synthesis with Fmoc protected amino acid derivatives built onto a TentaGelS NH_2_ resin bead support (Novabiochem, Iris Biotech GMBH). Repeated cycles of Fmoc deprotection and amide bond coupling were carried out using single N-protected amino acids followed by a final cleavage step to release the peptide sequence from the resin support.

The yield and purity of the peptide were examined using mass spectrometry and high performance liquid chromatography (HPLC). Mass spectrometry was carried out using a Bruker Daltronics MicrOTOF time-of-flight spectrometer set with a spray voltage of −4.5 KV, end point offset of −500 V, nebulizer gas pressure at 0.4 bar, dry gas at 4 L/min, and dry temperature of 180°C using a mass to charge ratio of 50 to 3000* m/z*. HPLC was carried out using a Phenomenex C18 column stationary phase with mobile phase HPLC grade water and acetonitrile gradient settings of water : acetonitrile 0.1 min 95% : 5%, 30 min 30% : 70%, 5 min 0 : 100%, and 2 min 95% : 5% and a flow rate of 0.8 mL/min.

All* in vitro* cell assays were carried out using donor tissue derived corneal cell strains in order to produce an isolated biological testing environment which more closely modelled the* in vivo* setting. Corneal tissue was obtained from MEH Lions Eye Bank, Moorfields Eye Hospital under ethics approval REC number 06/Q1907/81 using approved informed consent procedures for the use of human tissue in medical research. All ocular tissues were collected as corneoscleral rims.

For epithelial cell isolation, tissue sections of approximately 1 mm were removed from the limbal rings at the corneal/conjunctival junction and cut into small pieces prior to dissociation of cells in 0.5% trypsin (Gibco, UK) at 37°C for 40 minutes. Trypsin was inactivated by the addition of media and cells were centrifuged for 4 minutes at 400 G. The cell pellet was resuspended in media and cultured in 25 cm^2^ culture flasks or 24 well plates as required (Greiner Bio-One, UK). Cells were cultured at 37°C, 5% CO_2_ using R&G complete growth medium containing DMEM and Ham's F12 medium (Life Technologies, UK) at a 3 : 1 ratio, 10% heat inactivated foetal calf serum (FCS, GE Healthcare, UK), 10 ng/mL epidermal growth factor (EGF, Life Technologies, UK), 0.4 mg/mL hydrocortisone, and 10 nM cholera toxin on a feeder layer of lethally gamma-irradiated 3T3 feeder cells (2 × 10^4^ 3T3s per cm^2^) [33].

For keratocyte isolation, stromal tissue was dissected into small pieces and placed into 25 cm^2^ flasks. Stromal explants were incubated overnight at 37°C, 5% CO_2_ to allow attachment to the flask surface, and then submerged in DMEM supplemented with 10% FCS. All cells were grown until subconfluent and media were replaced every two to three days.

### 2.2. Assessment of Peptide Cytotoxicity Using MTS and LDH Assays

Cell viability was measured using the CytoTox 96 nonradioactive cytotoxicity assay and the CellTiter 96 AQ_ueous_ one solution cell proliferation assay (Promega, UK). Primary human keratocytes were used for these assays since these were more readily available than the LESCs. Cells were seeded into 96 well plates at 2.5 × 10^4^ cells per well and left to attach for 24 hours prior to addition of peptide in a range of concentrations from 0.01 to 1000 mg/mL for 24 hours. Analysis of cell proliferation and cytotoxicity was carried out according to manufacturer's standard protocols. 1% v/v Triton-X was used as a positive control. Gram-negative bacterial lipopolysaccharide (LPS from* Serratia marcescens*) was also assessed for cytotoxic effects using concentrations from 0.002 to 200 mg/mL.

### 2.3. Peptide Inhibition of Inflammatory Cytokine Production by Keratocytes and LESC

Infection and inflammation were assessed using isolated human keratocyte and LESC strains. Cells were stimulated with LPS to imitate cell response to bacterial infection and with IL-1*β* to imitate corneal inflammatory response, in which constitutively expressed and upregulated IL-1*β* is released into the extracellular space and underlying stroma following tissue damage. LPS and IL-1*β* stimulation of cytokines IL-8, IL-6, and IL-1*β* by corneal epithelial cells and keratocyte primary cell strains and the impact of IL-1ra peptide activity on this response were assessed.

Cytokine production by keratocytes in response to a range of concentrations of LPS (0–200 *μ*g/mL) and IL-1*β* (0 to 1 × 10^−9^ M, BD Biosciences, UK) was assessed in order to establish optimum concentrations to study the effect of the peptide. As availability of large stocks of LESC cultures was limited, studies using LESC were only performed using the optimum concentrations identified in experiments using keratocyte cultures (0.02 *μ*g/mL LPS and 1 × 10^−9^ M IL-1*β*).

Keratocytes at a concentration of 2 × 10^4^ cells were seeded into the wells of 96 well plates and allowed to attach for 6 hours. LESCs isolated from corneoscleral rims were seeded into the wells of 24 well plates and allowed to grow until subconfluent over 2 weeks. Cells were then incubated for 24 hours in serum-free medium spiked with LPS or IL-1*β*. 1% Triton-X, LPS/IL-1*β* only, and medium only groups were used as controls. Cell supernatants were collected and stored at −80°C prior to carrying out cytokine ELISAs according to manufacturer's instructions and using sample dilutions of 1 : 300 (IL-8/IL-6) and 1 : 10 (IL-1*β*) (BD Biosciences).

### 2.4. Keratocyte Expression of IL-1*β* mRNA following LPS Stimulation

Since no IL-1*β* protein was detected following stimulation of keratocytes with LPS, mRNA expression was assessed. Keratocytes (5 × 10^4^ cells per well) were cultured in 24 well plates for 6 hours prior to treatment in either medium only, 0.02 *μ*g/mL LPS, or 0.02 *μ*g/mL LPS plus 1 mg/mL peptide for 24 hours. RNA was extracted using a Qiagen RNeasy kit (Qiagen, Germany) and q-PCR was carried out using a Bio-Rad iScript cDNA synthesis kit (Bio-Rad, UK) and a Qiagen Rotor-Gene Q Pure Detection Machine to assess expression of IL-1*β* using isoform specific primer sets (Alta Biosciences, UK) [14]. All q-PCR assays were performed according to the MIQE guidelines [34].

### 2.5. Immunocytochemical Assessment of TLR4 Expression by Keratocyte and LESC Cultures

Since LPS stimulation induced upregulation of cytokine production by keratocytes and not by LESCs, the presence of TLR4 LPS receptors was assessed. Cells were cultured on 13 mm diameter cover slips, fixed with 3% v/v formaldehyde for 15 minutes, and permeabilized in perm/quench solution (50 mM ammonium chloride, 0.2% saponin) for 15 minutes. Cell were incubated in mouse anti-human TLR4 monoclonal antibody (AbCam, UK) diluted 1 : 50 in PGAS (0.2% gelatin from cold water fish skin, 0.02% saponin in PBS) for 1 hour in a humidified chamber followed by incubation with FITC conjugated goat anti-mouse IgG secondary antibody (AbCam, UK) diluted 1 : 100 in PGAS for 30 minutes at room temperature. Coverslips were mounted in Antifade Gold with DAPI reagent (Life Technologies, UK) and images were captured on a Zeiss Axioscope fluorescent microscope using AxioVision software (Zeiss Ltd, Germany). Secondary antibody only controls were used to assess primary antibody specificity.

### 2.6. Surface Modification, Functionalization, and Peptide Binding to Contact Lens

Silicon hydrogel based contact lens (Bausch & Lomb, UK) were surface modified with amine (NH_2_) groups. Each contact lens was submerged in 20% 3-aminopropyltriethoxysilane (APTES) for 15 minutes, washed in toluene and methanol, and then air-dried. The number of NH_2_ groups present on the functionalised contact lens surface was quantified using a methyl orange assay. Lenses were placed in the wells of a 24-well plate and incubated in methyl orange (476 *μ*M) at room temperature for 2 hours. Each lens was washed with 10 mM sodium phosphate, dried, and submerged in ethanol to remove any methyl orange bound to the lens. 100 *μ*L of this solution was transferred to a 96-well plate and absorbance was read at 490 nm using an ASYS UVH 340 plate reader. Concentrations were calculated using a standard curve of methyl orange ranging from 0 to 476 *μ*M. Since the methyl orange binds to NH_2_ groups with a 1 : 1 ratio, the concentration of methyl orange is proportional to the concentration of amine groups [35]. Nontreated lenses were used as controls.

The peptide carboxyl groups were cross-linked to the amine groups on the surface of each contact lens using 5 mM N-hydroxysuccinimide (NHS) and 2 mM 1-ethyl-3-(3′-dimethylaminopropyl)carbodiimide (EDC) prepared by dilution in 4-morpholinoethanesulfonic acid (MES) buffer (0.1 M of MES and 0.3 M of NaCl, pH 7). 1-2 mg peptide and 1 mL EDC/NHS buffer were incubated for 15 minutes at room temperature, added to the modified contact lens, and incubated for 2 hours at room temperature. Unbound peptide was removed by rinsing in PBS.

Successful binding of the peptide to the contact lens was measured using the bicinchoninic acid (BCA, Thermo Scientific, UK) assay. Peptide conjugated contact lenses were submerged in 300 *μ*L of 50 : 1 BCA/copper (II) sulphate solution and incubated at 37°C for 45 minutes. 200 *μ*L of the solution was transferred to a clean 96-well plate and absorbance was read at 562 nm. Concentrations of the peptide were calculated by comparison with a standard curve of known peptide concentrations. Nontreated lenses were included as controls.

### 2.7. Growth of LESC on Peptide-Conjugated Silicon Lenses

Corneal epithelial cells freshly isolated from 3 separate donors were seeded directly onto the peptide-conjugated contact lenses and compared with cells seeded onto glass coverslips. Cells were cultured in R&G medium and the phenotype of the cells was confirmed using immunocytochemical staining for K3 (Millipore, USA), K19 (Dako, UK), and p63 (Millipore, USA) (all at 1 : 50 dilution in PGAS) using the same method as described for TLR4 immunocytochemical staining in 2.5. K3 was used to identify corneal epithelial cells and K19 and p63 were used as putative markers of limbal epithelial stem cell fraction. Alexa Fluor 488 Phalloidin (Life Technologies, UK) (1 mg/mL in PBS) was used to observe cell structure. Secondary antibody only controls were used to assess primary antibody specificity.

### 2.8. Statistical Analysis

All experiments were performed with cells from at least three different donors. For statistical analysis, ANOVA all-pairwise comparison using the Holm-Sidak method was performed using SigmaStat (Systat, USA).

## 3. Results

### 3.1. Synthesis and Analysis of an IL-1 Receptor Antagonist Peptide

**Figure 1:**
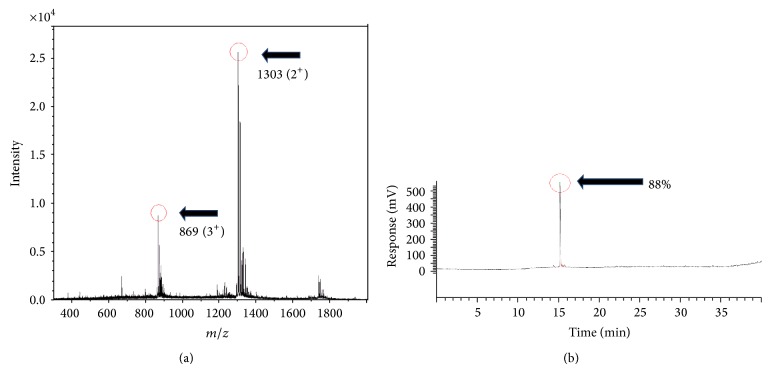
(a) Crude products from the synthesis of the IL-1ra analogue peptide (2606 Da) were analysed by mass spectrometry. Component intensity is shown on the *y*-axis in arbitrary units. Prominent peaks are at* m/z* 1303 (2^+^) and 869 (3^+^) and correspond to the original peptide molecular weight. (b) HPLC chromatogram of crude products from the synthesis of IL-1ra analogue peptide (MW 2606 Da) indicated a single prominent peak at 15-minute retention time.

Successful synthesis of the peptide was confirmed by MS and HPLC. Two prominent peaks were observed on the mass spectrum of the synthesised peptide. The most abundant peak was at* m/z* of 1303 (2+) and the second was at 869 (3+) corresponding with the correct molecular weight of the peptide at 2606 Da. The HPLC chromatogram of crude products from the synthesis of the peptide revealed a peak with retention time of 15 minutes indicating a purity yield of 88% (Figures 1(a) and 1(b)).

### 3.2. Assessment of Peptide Cytotoxicity Using MTS and LDH Assays

**Figure 2:**
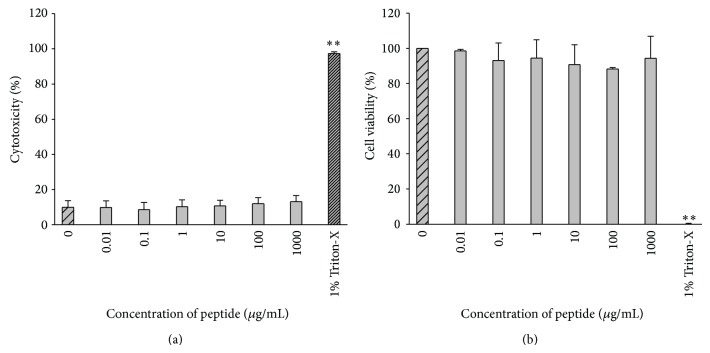
(a) LDH was measured to assess the cytotoxic effect of keratocyte treatment with increasing concentrations of peptide (0.01–1000 *μ*g/mL). (b) MTS conversion to formazan was measured to assess peptide impact on keratocyte metabolism treated as in (a). No significant difference was shown in peptide treated groups compared to nontreated controls (*P* > 0.05) (*n* = 3, mean ± sd).

The peptide was not cytotoxic to keratocytes at a concentration range of up to 1000 *μ*g/mL and had no significant effect on cell viability (Figure 2) (*P* < 0.01, *n* = 3). LPS was not cytotoxic to keratocytes but induced a significant reduction in cell viability to 80% at a concentration of 200 *μ*g/mL.

### 3.3. Peptide Inhibition of Inflammatory Cytokine Production by Corneal Cells Stimulated with LPS or IL-1*β*

**Figure 3:**
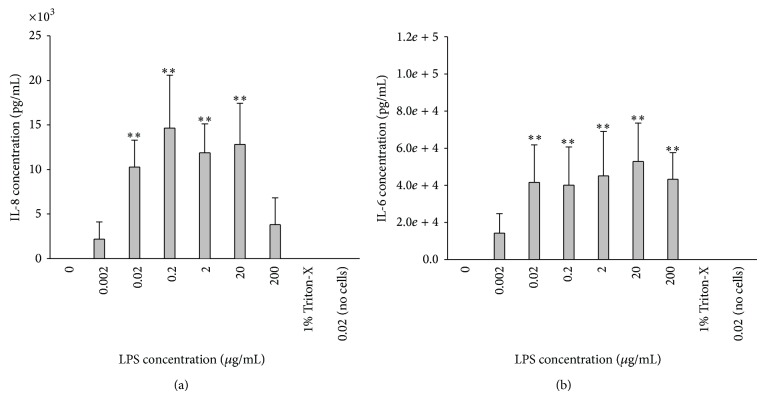
IL-6 and IL-8 production by keratocytes in response to increasing concentrations of LPS. LPS (0.02–200 *μ*g/mL) induced a significant rise in (a) IL-8 and (b) IL-6 secretion by keratocytes when compared to the non-LPS treated controls (^**^ *P* < 0.01). In both assays controls of Triton-X and 0.02 *μ*g/mL LPS (no cells) were included (*n* = 6, mean ± sd).

**Figure 4:**
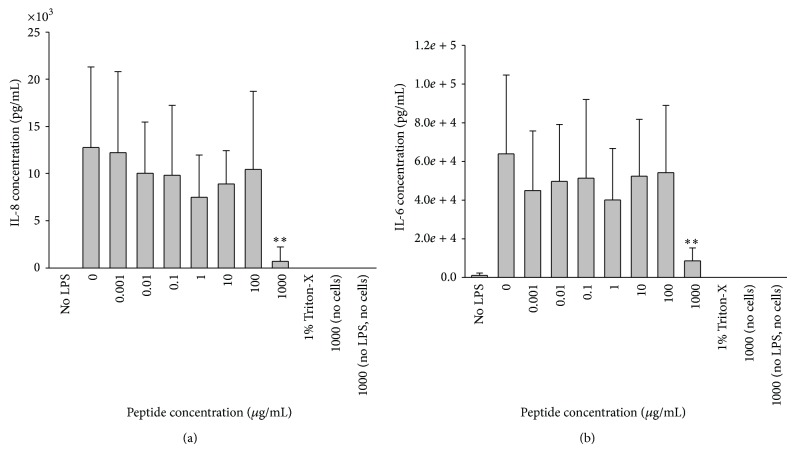
Peptide suppression of IL-6 and IL-8 production by LPS-stimulated keratocytes. (a) IL-8 and (b) IL-6 were secreted by keratocytes when stimulated with 0.02 *μ*g/mL LPS and a range of peptide concentrations (0.001–1000 *μ*g/mL). The addition of 1000 *μ*g/mL peptide resulted in a significant reduction in cytokine secretion (^**^ *P* < 0.01). (*n* = 6, mean ± sd). Peptide (1000 *μ*g/mL) with no cells, peptide (1000 *μ*g/mL) and LPS (0.02 *μ*g/mL) without cells, and Triton-X and cells were included as controls.

Keratocytes secreted up to 20 ng/mL IL-8 following stimulation with LPS at a concentration range of 0.02 to 20 mg/mL and up to 75 ng/mL IL-6 following stimulation with 0.02 to 200 mg/mL (Figure 3). Keratocytes did not secrete detectable IL-1*β* in response to LPS stimulation. Addition of peptide at concentrations ranging from 1 ng/mL to 1000 *μ*g/mL induced a significant decrease in production of IL-8 and IL-6 to negligible levels at the highest concentration of 1000 *μ*g/mL (Figure 4). Stimulation of LESC with LPS did not yield any detectable IL-8, IL-6, or IL-1*β* secretion.

**Figure 5:**
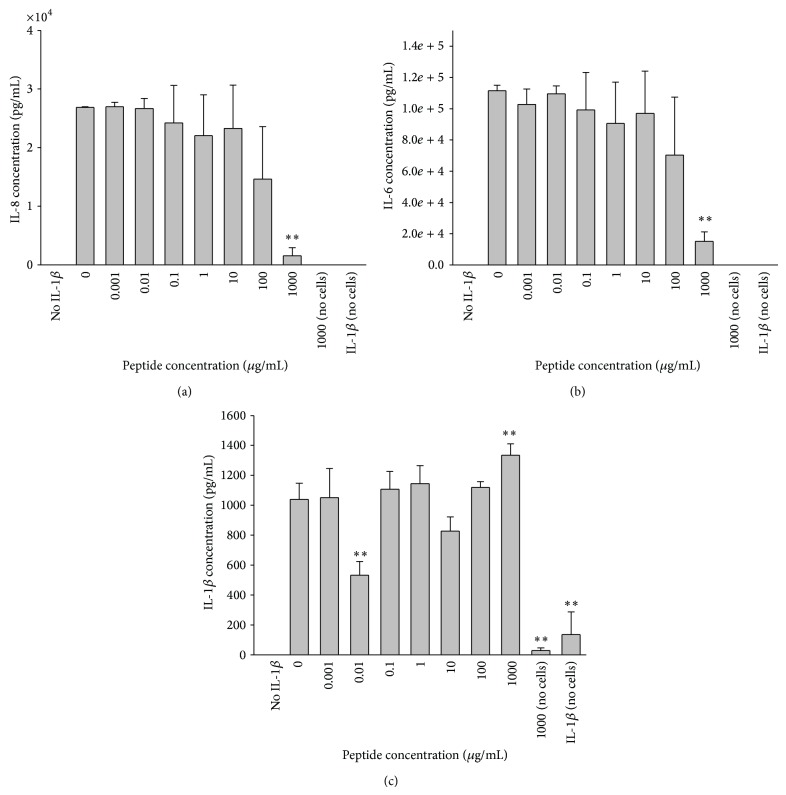
Peptide suppression of cytokine production by IL-1*β* stimulated keratocytes. (a) IL-8 and (b) IL-6 secretion by keratocytes stimulated with IL-1*β* (10^−9^ M) and a range of peptide concentrations (0.001–1000 *μ*g/mL) was significantly reduced following addition of 1000 *μ*g/mL peptide when compared to the group containing no peptide (^**^ *P* < 0.01). (c) IL-1*β* secretion by keratocytes stimulated with IL-1*β* (10^−9^ M) and a range of peptide concentrations (0.001–1000 *μ*g/mL) showed significant differences in cytokine secretion at 0.01 *μ*g/mL and 1000 *μ*g/mL (^**^ *P* < 0.01) compared to the no peptide added controls (*n* = 3, mean ± sd).

**Figure 6:**
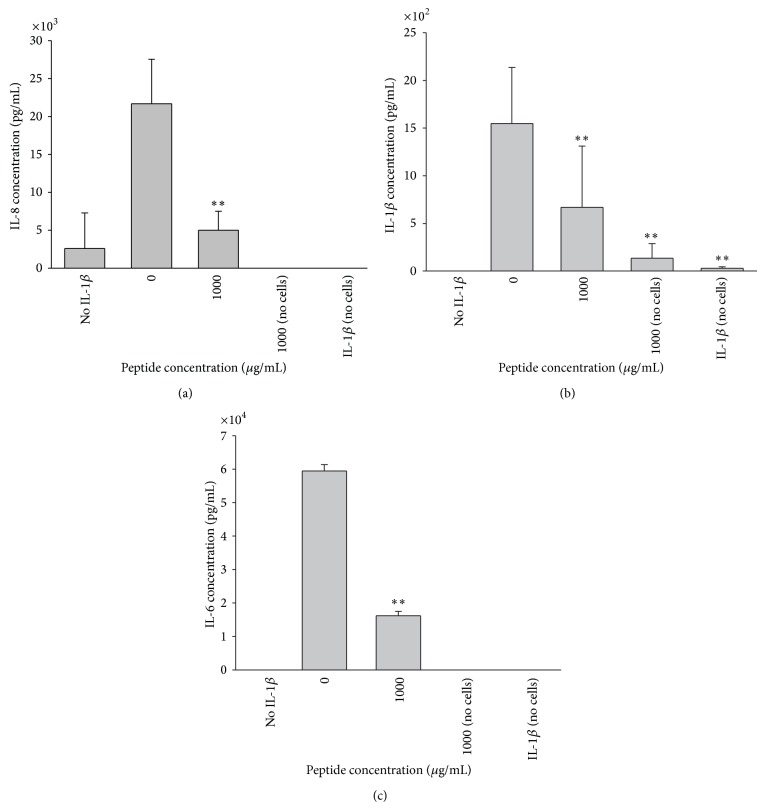
Peptide suppression of cytokine production by IL-1*β* stimulated LESC. (a) IL-8, (b) IL-6, and (c) IL-1*β* secretion by LESC when stimulated with IL-1*β* (10^−9^ M) were significantly reduced following the addition of peptide (1000 *μ*g/mL) compared to the no peptide controls (^**^ *P* < 0.05) (*n* = 3, mean ± sd).

When keratocytes were stimulated with recombinant IL-1*β* the addition of the peptide at a concentration of 1000 *μ*g/mL resulted in a statistically significant decrease in IL-8 and IL-6 (*P* < 0.01) (Figures 5(a) and 5(b)). A significant reduction in IL-1*β* levels occurred when the peptide was added at a concentration of 0.01 *μ*g/mL, while a significant increase was observed at 1000 *μ*g/mL (Figure 5(c)). Keratocytes are known to upregulate IL-1*β* via an autocrine loop and a number of different stimuli so that the IL-1*β* response to peptide inhibition does not follow the same pattern of inhibition as for IL-6 and IL8 [36–38]. After stimulation of LESC with IL-1*β*, addition of 1000 *μ*g/mL peptide resulted in a significant reduction in IL-8, IL-6, and IL-1*β* levels (Figure 6).

These results show that the IL-1ra peptide analogue was able to inhibit the production of proinflammatory cytokines IL-6, IL-8, and IL-1*β* in both LESC and keratocytes following stimulation with recombinant IL-1*β* and was able to inhibit the production of IL-6 and IL-8 by keratocytes following LPS stimulation.

### 3.4. Keratocyte Expression of IL-1*β* mRNA following LPS Stimulation

**Figure 7:**
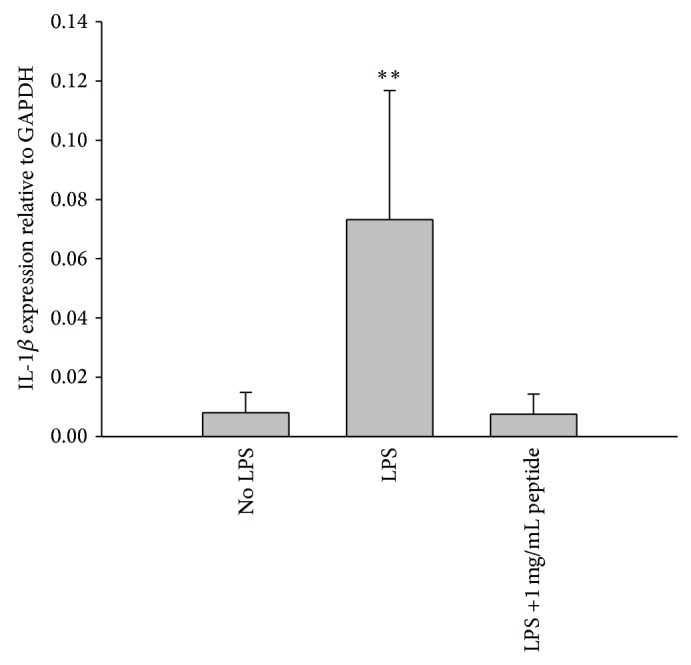
IL-1*β* gene expression in LPS stimulated keratocytes treated with peptide was compared to LPS stimulated, nonpeptide treated, and non-LPS stimulated controls using q-PCR. Relative genetic expression of IL-1*β* was compared to expression of the GAPDH housekeeping gene. Keratocytes stimulated with LPS demonstrated a significant increase in IL-1*β* gene expression compared to the non-LPS stimulated control. The addition of 1000 *μ*g/mL peptide significantly reduced IL-1*β* expression to that of the non-LPS stimulated control (^**^ *P* < 0.05) (*n* = 3, mean ± sd).

Although IL-1*β* secretion by keratocytes could not be detected by ELISA following LPS stimulation, a significant increase in IL-1*β* mRNA levels was detected by q-PCR. This was significantly reduced when 1 mg/mL peptide was added (Figure 7) indicating that LPS and peptide do have an effect on IL-1*β* levels in keratocytes, although levels may be below the detection limit of ELISA.

### 3.5. Immunocytochemical Assessment of TLR4 Expression by Keratocyte and LESC Cultures

**Figure 8:**
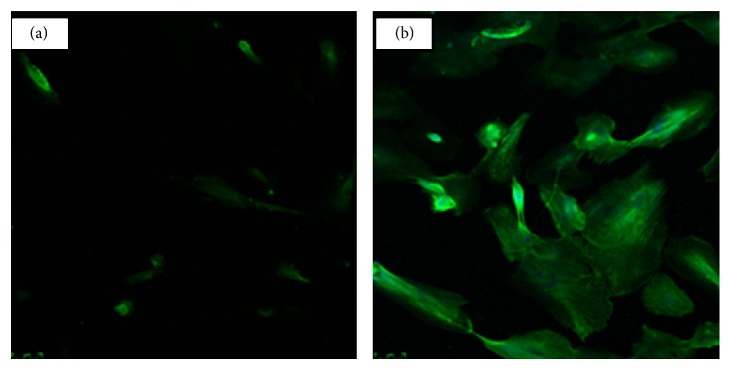
Immunocytochemical staining of TLR4 in (a) nonpermeabilized keratocytes and (b) permeabilized keratocytes (mag ×200) No TLR4 was detected in LESCs (data not shown).

Since LPS stimulation resulted in cytokine upregulation in keratocytes but not in LESC, the presence of toll-like receptor 4 (TLR4), which mediates LPS activity, was assessed by immunocytochemistry. TLR4 could be readily detected in the cytoplasm of permeabilized keratocytes, indicating a cytoplasmic pool of the protein, as well as on the plasma membrane of nonpermeabilized cells, further indicating active TLR-4 receptor (Figure 8). However, no staining could be detected in the cytoplasm or cell membrane of LESC explaining the lack of cytokine upregulation by LPS in LESC.

### 3.6. Surface Modification, Functionalization, and Peptide Binding to Contact Lens

**Figure 9:**
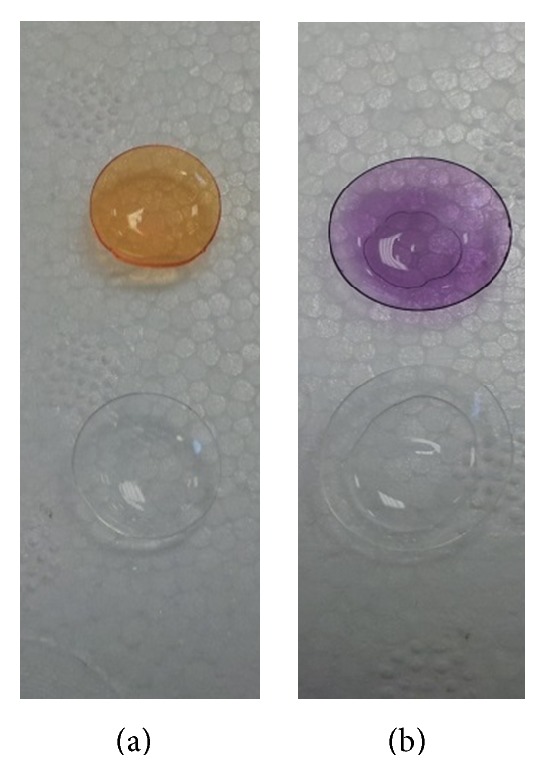
Functionalization of the silicone hydrogel contact lenses: (a) methyl orange assay of APTES treated (top) and untreated (bottom) lenses showing the presence of NH_2_ groups in the APTES treated lens and (b) BCA assay of peptide treated (top) and untreated (bottom) lenses showing the presence of peptide on the top lens.

Successful functionalization of the lens surfaces with NH_2_ groups was required for peptide binding and was confirmed using methyl orange. Treatment with APTE and methyl orange resulted in distinctive orange colouration, indicating the presence of NH_2_ groups, whilst untreated lenses remained colourless (Figure 9(a)). An average NH_2_ binding of 66.5 nmol was observed through calculation of methyl orange at a 1 : 1 binding ratio of dye to NH_2_ groups. The BCA assay was used to measure the amount of peptide bound to the contact lens surface. Lenses treated with APTES and peptide stained purple indicating the presence of peptide. Untreated lenses remained colourless (Figure 9(b)). Adding 1000 *μ*g/mL (115 nM) peptide to the APTES treated lenses resulted in average binding of 2.7 nmol peptide. Increasing the peptide concentration did not increase the amount of peptide bound to the contact lens indicating that the NH_2_ groups on the surface are the limiting factor.

### 3.7. Growth of LESCs on Peptide-Conjugated Lenses

**Figure 10:**
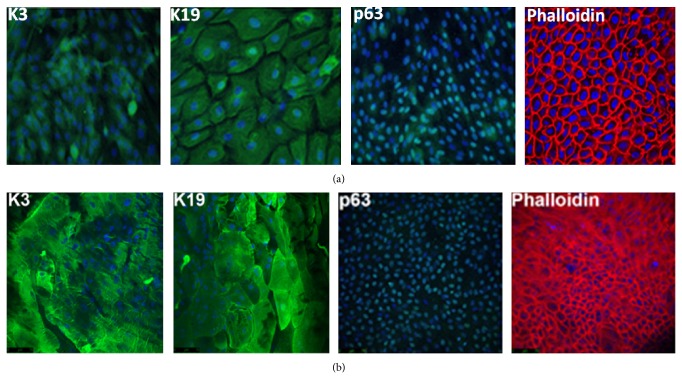
Immunocytochemical K3, K19, and p63 staining of primary LESC cultures cultured on (a) glass coverslips and (b) functionalised contact lenses. All corneal epithelial cells and putative LESC markers (K3, K19, and p63) were present indicating the presence of limbal epithelial cells on the functionalised contact lens. The curve of the contact lens can be seen for images in (b). The tightly packed, cobblestone morphology characteristic of epithelial cells can be observed following cytoskeletal staining with phalloidin. Cell nuclei were stained with DAPI (Leica confocal microscope, mag ×200).

LESCs were successfully cultured on the inner surface of the peptide modified contact lens without affecting LESC morphology and phenotype compared to cells grown on glass coverslips (Figures 10(a) and 10(b)). Phalloidin stain showed characteristic epithelial morphology. The presence of LESC markers cytokeratin 3 (K3), cytokeratin 19 (K19), and p63 further confirmed normal LESC phenotype.

## 4. Discussion

IL-1 is considered to be a “master” cytokine in the eye, due to its involvement in the initial response to injury or insult [39] as well as its ability to stimulate the production of other proinflammatory cytokines [40, 41]. The mechanism of action of IL-1 is key to inflammatory responses in the eye, facilitated by its constitutive expression by corneal epithelial cells and the presence of IL-1 receptor on keratocytes in the underlying stroma [42]. Thus, any breakdown of the corneal surface or penetrating injury to the cornea results in the release of IL-1 and subsequent proinflammatory signalling and upregulation of IL-6, IL-8, and more IL-1 in underlying keratocytes [43, 44]. In cases of LSCD, patients often present with persistent epithelial breakdown and chronic stromal inflammation [11, 45], associated with the recruitment of elevated numbers of leukocytes to the normally immune privileged cornea [12, 46]. This influx of leukocytes has been linked to increased possibility of rejection of allografts used to repair the damaged cornea [47]. Therefore, a reduction in proinflammatory IL-1 signalling may improve management and control of corneal inflammation and result in improved graft survival. The use of recombinant IL-1ra has previously been investigated as a possible means of attenuating IL-1 signalling in the cornea [48, 49]. However, recombinant proteins have prohibitive costs [50] and are less able to bind to surfaces in conformationally active form because of their size. As a result we have chosen to investigate the use of short amino acid chain peptides to mimic the function of IL-1ra and assess the feasibility of combining this peptide with a contact lens carrier.

A 21 amino acid peptide IL-1ra analogue, based on the work of Yanofsky et al. and Vigers et al. [31, 32], was produced and tested for potential anti-inflammatory properties using an* in vitro* model of corneal inflammation. The long primary structure of the peptide increases the risk of improper structural folding and mismatching of amino acids during production. However by application of repeated coupling steps for each amino acid in the peptide sequence during production, the peptide was successfully synthesised with a purity of 88% as shown by HPLC. Potential peptide toxicity was measured by standard MTS and LDH assays since peptide synthesis involves reagents that may be toxic [51]. Toxicity can cause side effects such as corneal melting similar to that seen in treatment with NSAIDs [52] and inflammatory stimulants may induce cytotoxic effects [53]. The peptide was not cytotoxic to keratocytes up to the highest test concentration of 1 mg/mL.

We first tested our model of corneal inflammation. A minimum concentration of 0.02 *μ*g/mL LPS was required to induce measurable cytokine production by keratocytes following 24 hours of incubation. This time period was selected based on work by Shtein et al. suggesting that keratocyte cytokine production is optimal following LPS stimulation over 24 hours [47].

Contrary to work published by others, LPS stimulation did not result in cytokine production by LESC, despite utilising the same source of LPS as previously reported [54–58].

LPS induces downstream signalling and inflammation in cells through binding to toll-like receptors (TLRs), especially TLR4 [59]. TLR4 has been reported to be absent from the plasma membrane of epithelial cells, where it is expressed only intracellularly [59, 60]. In our study, TLR4 was indeed absent from the plasma membrane of LESC. This is perhaps not surprising, as the corneal epithelium acts as an external barrier to the outside environment and is thus regularly exposed to environmental pathogens. An inflammatory response to regular contact with bacterial-derived LPS may therefore be unnecessary and inappropriate. When damage to the surface of the cornea occurs and such environmental pathogen products are able to enter the cells, activation of intracellular pathways to inflammation occurs. The LESC strains used in our study were derived from at least 3 different donors for the experiments to allow for donor variability in LPS response. Our data showing the absence of TLR expression in the LESCs explains the absence of cytokine response to LPS.

LPS was able to stimulate IL-8 and IL-6 production, but IL-1*β* was only measurably increased at the mRNA level (*P* < 0.01). The results indicate that keratocytes do upregulate IL-1*β* gene expression upon LPS stimulation but may require further stimulants for translation and secretion or may require a longer time frame.

In addition to LPS, we tested the ability of IL-1*β* to stimulate inflammatory cytokine production in our inflammatory model. IL-1*β* was able to stimulate significant production of IL-8, IL-6, and IL-1*β* in both cell types. We next tested the ability of the recombinant peptide to reduce proinflammatory signalling in our model.

A peptide concentration of 1 mg/mL was able to significantly reduce the release of IL-8 and IL-6 from keratocytes stimulated with LPS. Furthermore, the same concentration of peptide was able to suppress IL-1*β* gene expression. When keratocytes and LESCs were stimulated with IL-1*β*, the peptide was shown to significantly reduce the release of IL-8 and IL-6 in both cell types and IL-1*β* in LESC. The response in keratocytes was variable which is likely due to the autocrine feedback signalling loop in these cells which results in IL-1*β* upregulation [36–38].

LSCD can occur when insult to the cornea penetrates the epithelial layers to the underlying stroma, allowing IL-1*β* released by epithelial cells to subsequently stimulate keratocytes in the stroma promoting inflammation, through inflammatory mediators IL-8 and IL-6. Reduction of these cytokines on peptide addition suggests that peptide use may lead to a reduction in inflammation and improved integration of allografts used to repopulate the corneal surface.

Communication between cells in the stromal and epithelial layers and a balanced inflammatory response is important in wound healing [36, 37, 61]. For example, IL-1*β* stimulation of keratocytes results in upregulation of HGF and KGF, which has been shown to improve reepithelialisation [62–64]. The peptide utilised in this study reduced inflammatory cytokine release by both keratocytes and LESC significantly.However, measurable levels of cytokine were present indicating a baseline inflammatory response in the presence of peptide but the potential to suppress the peaks of excessive inflammatory cytokine production during chronic inflammation.

In order to develop a delivery system suitable for clinical application, LESCs were grown on a carrier, which was functionalised with the peptide. Bausch & Lomb PureVision (balafilcon A) contact lenses were used since they are already approved for 30-day extended wear and therapeutic use, allowing sufficient time for the transplanted LESC to repopulate the epithelial surface. Contact lenses have previously been used in ocular surface reconstruction. Di Girolamo et al. [65] cultured corneal cells directly onto a contact lens for patients to wear for a time period of between 14 and 22 days. Successful reestablishment of the ocular surface was demonstrated following 13 months postsurgical follow-up. When amnion is utilised as a carrier, an average time of 22.8 days has been reported to treat partial LSCD [66]. These studies suggest that 30-day continuous wear will provide a sufficient timeframe for LSCD treatment. Functionalization of the lens, by establishing amine groups to facilitate peptide binding, resulted in successful binding of 2.7 nmol peptide to the inner surface and successful growth of LESC sheets on the surface of the lens. Results suggest that a surface functionalised peptide or eluted peptide fraction could be used to effectively mimic the anti-inflammatory function of amniotic membrane in part by reducing IL-1 proinflammatory signalling in order to improve graft integration and clinical outcome.

## 5. Conclusion

Whilst the role of topical Il-1*β* antagonist has previously been demonstrated in the control of corneal inflammation, our work is the first to synthesise and characterise a short chain IL-1 receptor antagonist peptide for this application using isolated corneal cell cultures to assess peptide suppression of inflammatory pathways and a functionalised silicon hydrogel carrier to assess the effect of peptide coating on corneal epithelial stem cell growth. Use of a short chain peptide rather than full length protein is cost-effective and improves conformational binding of active peptide to a carrier surface. The peptide suppressed TLR4 mediated LPS stimulation of cytokine production in keratocytes and IL1*β* stimulated cytokine production in limbal epithelial stem cells and keratocytes. Peptide coated silicon hydrogels were able to maintain limbal epithelial stem cell fraction and support epithelial sheet growth. The peptide may be used to augment corneal epithelial sheet delivery for grafting and to control excessive inflammation which limits current grafting success.
